# Procalcitonin as a marker of Candida species detection by blood culture and polymerase chain reaction in septic patients

**DOI:** 10.1186/1471-2253-14-9

**Published:** 2014-02-21

**Authors:** Andrea Cortegiani, Vincenzo Russotto, Francesca Montalto, Grazia Foresta, Giuseppe Accurso, Cesira Palmeri, Santi Maurizio Raineri, Antonino Giarratano

**Affiliations:** 1 Department of Biopathology, Medical and Forensic Biotechnologies (DIBIMEF), Section of Anaesthesia, Analgesia, Intensive Care and Emergency. Policlinico "P. Giaccone", University of Palermo, Palermo, Italy

**Keywords:** Procalcitonin, Sepsis, Candida species, Blood stream infection, Fungal infection, Polymerase chain reaction, Antifungal therapy

## Abstract

**Background:**

The aim of our study is to test procalcitonin (PCT) as surrogate marker of identification of Candida spp. by blood culture (BC) and real-time-polymerase chain reaction (PCR), whether alone or in association with bacteria, in septic patients.

**Methods:**

We performed a single-centre retrospective study. We reviewed the clinical charts of patients with a diagnosis of severe sepsis or septic shock treated at our general intensive care unit from March 2009 to March 2013. We analysed all diagnostic episodes consisting of BC, real-time PCR assay and dosage of PCT. We registered age, sex, white blood count, sequential organ failure assessment score and type of admission between medical or surgical. When inclusion criteria were met more than once, we registered the new diagnostic episode as subsequent diagnostic episode. The diagnostic performance of PCT to predict Candida spp. identification alone or in mixed infections by either BC or PCR was tested using the receiver-operative characteristic curve. Logistic regression was constructed using presence of Candida spp. as the dependent variable.

**Results:**

A total of 260 diagnostic episodes met the inclusion criteria. According to BC results classification, a significantly lower value of PCT was observed in Candida spp. BSI (0.99 ng/ml, 0.86 - 1.34) than in BSI caused by bacteria (16.7 ng/ml, 7.65 - 50.2) or in mixed infections (4.76 ng/ml, 2.98 - 6.08). Similar findings were observed considering PCR results. A cut-off of ≤ 6.08 ng/ml for PCT yielded a sensitivity of 86.8%, a specificity of 87.4%, a positive predictive value of 63.9%, a negative predictive value (NPV) of 96.3% and an area under the curve of 0.93 for Candida spp. identification by BC. A similar high NPV for a cut-off ≤ 6.78 ng/ml was observed considering the classification of diagnostic episodes according to PCR results, with an AUC of 0.85. A subsequent diagnostic episode was independently associated with Candida spp. detection either by BC or PCR.

**Conclusion:**

PCT could represent a useful diagnostic tool to exclude the detection of Candida spp. by BC and PCR in septic patients.

## Background

Candida species (spp.) are currently among the leading microorganisms causing bloodstream infection (BSI) in critically ill patients worldwide. They are responsible for high crude mortality and healthcare costs [1-3]. A delay in starting adequate antifungal treatment is an independent predictor of high in-hospital mortality [4-6]. Nonetheless, BC requires several days for Candida spp. detection and it is still the gold standard for microbiological diagnosis [7-9]. β-1,3-D-glucan and mannan antigen in association with anti-mannan antibodies have been recently recommended as surrogate markers of Candida spp. infection [7,10]. However, their use has several limitations [7,11-15]. Polymerase chain reaction (PCR) is an alternative method to promptly identify DNA of microorganisms but its diagnostic performance in fungal infections is still questioned [7,16,17]. Procalcitonin (PCT) is widely used as marker of bacterial infection [18-20] and as guide to reduce patients’ exposure to antibiotics [21]. Few data support its usefulness as a surrogate marker for Candida spp. infection [22-24]. However, its value in patients with a mixed infection sustained by both bacteria and Candida spp., have not been investigated. Furthermore, no studies have described the correlation between PCT value and identification of Candida spp. DNA by PCR. The aim of our study is to test PCT as surrogate marker of Candida spp. detection by BC or PCR whether alone or in association with bacteria in septic patients.

## Methods

We obtained the approval from our institutional review board (Comitato Bioetico AOUP "P. Giaccone", Palermo, Italy) for this single - centre retrospective study. Informed consent was waived due to the retrospective and anonymous nature of data collection and analysis. We reviewed the clinical records of all patients with a diagnosis of severe sepsis or septic shock [25] from March 2009 to March 2013 at the general Intensive Care Unit of the University Hospital "P. Giaccone", Palermo, Italy. We analysed the diagnostic episodes performed after the diagnosis of severe sepsis or septic shock and registered those in agreement with our diagnostic protocol consisting of a blood culture, a PCR assay for microorganism detection (bacteria and/or Candida spp.) and a dosage of PCT. We excluded the diagnostic episodes done on patients with known immunodeficiency not related to sepsis, those with missing tests and those not completed within a 8-hour time interval. For each complete diagnostic episode, we registered age, sex, white blood count (WBC), Sequential Organ Failure Assessment (SOFA) score [26] and type of admission of the corresponding patient. The admission was considered as surgical if patient underwent surgery within 30 days preceding the ICU admission, otherwise it was considered as medical. According to the results of each microbiological test, the diagnostic episodes were then classified in four groups: bacterial infection, mixed infection (bacterial infection and Candida spp. infection), Candida infection and negative. We also subdivided the bacterial and mixed BSI according to Gram staining. When inclusion criteria were met more than once for a single patient during the ICU admission (i.e. new BSI documented by BC and PCR completed by a PCT determination), we considered this event a new diagnostic episode for data analysis. Nevertheless, we registered the event as subsequent diagnostic episode*.* In our ICU, a set of three blood specimens is drawn from each patient for BC, according to the recently published guidelines of the European Society of Clinical Microbiology and Infectious Diseases (ESCMID) [7]. We considered only blood specimens collected through venipuncture. A blood specimen of 5 ml is collected in a EDTA test tube for PCR analysis. We excluded the diagnostic episode from the analysis if a violation of the protocol for sterile procedures was encountered during clinical records review. In our institution, BACTEC® 9050 system (Becton Dickinson Diagnostic Instrument System, Paramus, NJ, USA) is used for the first step of blood culture process. A multiplex real time PCR (LightCycler Septifast®, MGRADE®, Roche Molecular Diagnostics, Prague, Czech Republic) is adopted as additional diagnostic tool for detection of the 25 most common pathogens causing BSI in our general ICU. This PCR assay is able to identify 5 Candida species (albicans, parapsilosis, tropicalis, krusei, glabrata). PCT is dosed by immunoassay through the Kriptor® PCT (Brahms, Hennigsdorf, Germany). Primary outcome of the study was to assess PCT median values in septic patients with a BSI caused by Candida spp. and/or bacteria detected by BC/PCR. Secondary outcome was the evaluation of the diagnostic performance of PCT with the identification of its sensitivity, specificity, positive predictive value (PPV) and negative predictive value (NPV) for detection of Candida spp.

### Statistical analysis

Variables distribution was analyzed by D’Agostino-Pearson’s test. For variables with normal distribution, we calculated and reported mean and standard deviation. Variables without a normal distribution were expressed as median and interquartile ranges (25th-75th) and comparisons were performed through Mann–Whitney U-test. One-way-analysis of variance ANOVA was used to test the difference between the means of different subgroups variables. Prior to ANOVA test, Levene’s test for a quality of variances was performed. If the ANOVA test was positive, we performed a Student-Newman-Keuls test or pairwise comparison of subgroups. The Kruskall-Wallis test (H-test) was used to analyse the effect of a classification factor on ordinal data when the distribution of the sample was not normal. For post-hoc analysis, if the Kruskall-Wallis test was positive, we performed a test for pairwise comparison of subgroups according to Conover [27]. A frequency table was constructed and the chi-square test was adopted for comparison of proportions. The diagnostic performance of PCT to predict the identification of Candida spp. by either BC or PCR was tested using the receiver-operating characteristic (ROC) curves. We calculated the area under the ROC curves (AUC) and the values with the highest Youden index (best PCT cut-offs). Logistic regression analysis was performed with presence of Candida spp. (Candidemia) as the dependent variable whether alone or in association with bacteria. The power of the calculated model was tested by Hosmer - Lemeshow goodness of fit test. A variable was entered into the model if its associated significance level was p < 0.05. On the contrary, variables were removed from the model if p > 0.1. Then, significant variables were sequentially entered into the model, checking and possibly removing variables as they became not significant (stepwise method). Regression coefficients (β coefficient) were calculated and reported. A value of p < 0.05 was considered statistically significant. Statistical analysis was performed using MedCalc for Windows, version 9.5.0.0 (MedCalc Software, Mariakerke, Belgium).

## Results

**Figure 1:**
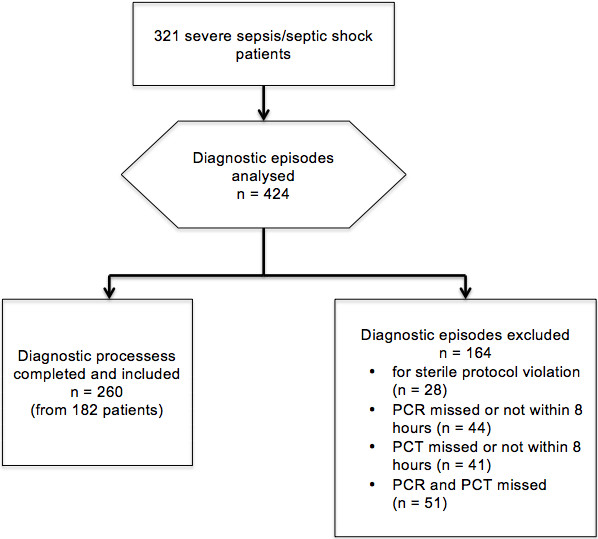
Flow-chart of the study.

A total of 321 severe sepsis/septic shock patients were admitted to our ICU during the study period, accounting for a total of 424 diagnostic episodes. Among them, 260 fulfilled the adopted inclusion criteria (Figure 1). BC detected 151 BSI caused by bacteria (18 caused by Gram +, 32 by Gram + and Gram -, 101 by Gram -), 22 by Candida spp., 31 mixed infection sustained by both bacteria and Candida spp. (5 Candida spp. and Gram +, 26 Candida spp. and Gram -) and 56 negative specimens. According to PCR results, 143 BSI were caused by bacteria spp., (25 caused by Gram +, 27 by Gram + and Gram -, 91 by Gram -), 30 by Candida spp., 39 by a mixed infection (8 Candida spp. and Gram +, 31 Candida spp. and Gram -) and 48 were negative (Table 1).

**Table 1:** Diagnostic episodes classification according to BC and PCR results

**Blood culture results**					
	Bacteria	Mixed	Negative	Candida	Total
**N°**	151	31	56	22	260
**PCR results**					
	Bacteria	Mixed	Negative	Candida	Total
**N°**	143	39	48	30	260

For differentiation of Bacteria according to Gram staining, see text.

**Figure 2:**
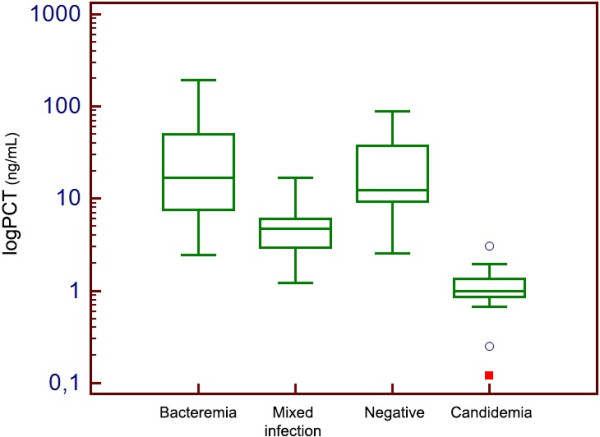
Box-plot distribution of PCT values according to BC classification of diagnostic episodes.

**Figure 3:**
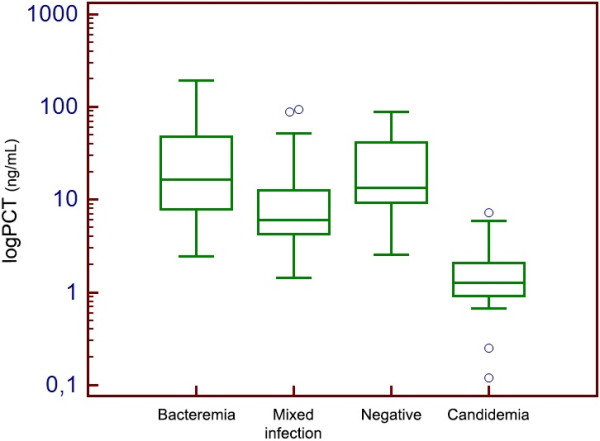
Box-plot distribution of PCT values according to PCR classification of diagnostic episodes.

Demographic data and characteristics of all diagnostic episodes are showed in Tables 2 and 3 for BC and PCR results respectively. A higher proportion of surgical patients was detected in Candida spp. BSI than in those caused by bacteria (p = 0.05) or negative (p = 0.03) according to BC results. With regard to infections demonstrated by BC, a higher percentage of subsequent diagnostic episodes was encountered in Candida spp. BSI and mixed BSI (p = 0.0001). Similar results were found according to PCR classification. PCT values are showed in Table 4 for BC and in Table 5 for PCR results. Considering BC results, a significantly lower value of PCT was observed in Candida spp. BSI (0.99 ng/ml, 1.34 - 0.86) than in BSI caused by bacteria (16.7 ng/ml, 50.2 - 7.65) or in mixed infections (4.76 ng/ml, 6.08 – 2.98) (Figure 2). Similar findings were observed when diagnostic episodes were classified according to PCR results (Figure 3).

**Table 2:** Demographic data for BC diagnostic episodes

	**Bacteremia (1)**	**Mixed (2)**	**Negative (3)**	**Candida (4)**	**Overall**	**P value**
**Diagnostic episodes**	151	31	56	22	260	
**Age** Mean (SD)	65.3 (13.9)	67.1 (14.1)	65.2 (13.4)	68.8 (11.7)	65.8 (13.6)	0.66
**Sex (male) (%)**	79 (52.3)	17 (54.9)	32 (57.1)	11 (50)	139 (53.5)	0.92
**WBC** (cells x10^3^/μL) Median (I.Q)	16.1 (14.1-18.2)	17.2 (14.2-18.3)	16.1 (13.0-18.1)	16.9 (14.9 -18.5)	16.2 (13.9-18.2)	0.24
**SOFA** Mean (SD)	7.8 (1.4)	7.9 (2.1)	7.6 (1.1)	8.2 (1.6)	7.8 (1.5)	0.5
**Surgical (%)**	90 (59.6)	20 (64.5)	29 (51.8) 3 vs 4 p = 0.03	19 (86.4) 1 vs 4 p = 0.05	158 (60.8)	0.04
**Septic Shock (%)**	52 (34.4)	9 (29)	15 (26.8)	7 (31.8)	83 (31.9)	0.74
**Subsequent Diagnostic Episode (%)**	40 (26.5)	21 (66.7) 1 vs 2 p = 0.0057	15 (26.8) 3 vs 4 p = 0.0048	18 (81.8) 1 vs 4 p = 0.0003	94 (36.2)	0.0001

ANOVA test for comparisons of means. Kruskall – Wellis test for comparisons of medians.

*SD* standard deviation, *I.Q.* interquartile range.

**Table 3:** Demographic data for PCR diagnostic episodes

	**Bacteremia (1)**	**Mixed (2)**	**Negative (3)**	**Candida (4)**	**Overall**	**P value**
**Diagnostic episodes**	143	39	48	30	260	
**Age** Mean (SD)	65.2 (13.8)	67.1 (14.3)	64.3 (13.1)	69.2 (11.3)	65.8 (13.5)	0.37
**Sex (male)** (%)	74 (51.7)	20 (51.3)	28 (58.3)	16 (53.3)	138 (53.1)	0.87
**WBC** (cells x10^3^/μL) Median (I.Q)	16.1 (13.9 -18.2)	16.9 (14.4-18.2)	16.4 (13.0 -18.1)	16.7 (14.2 -18.4)	16.2 (13.9-18.2)	0.58
**SOFA** Mean (SD)	7.8 (1.4)	8.1 (2.0)	7.6 (1.1)	8.0 (1.4)	7.8 (1.5)	0.3
**Surgical (%)**	87 (60.8)	22 (56.4)	25 (52.1)	24 (80.0)	158 (60.8)	0.09
**Septic Shock (%)**	52 (36.4)	9 (23.1)	15 (31.2)	7 (23.3)	83 (31.9)	0.29
**Subsequent Diagnostic Episode %**	38 (26.6)	23 (58.9)1 vs 2 p = 0.02	14 (29.2)	19 (63.3) 1 vs 4 p = 0.02	94 (36.2)	0.0001

ANOVA test for comparisons of means. Kruskall – Wellis test for comparisons of medians.

*SD* standard deviation, *I.Q.* interquartile range.

**Table 4:** Procalcitonin values according to the classification of BC results

**Group**	**PCT (ng/ml) Median (I.Q.)**	**Different (p < 0.05) from**
**Bacteremia**	16.75 (7.65 - 50.5)	Mixed, Candidemia
**Mixed**	4.76 (2.98 - 6.08)	Bacteremia, Negative, Candidemia
**Negative**	12.36 (9.36 - 37.2)	Mixed, Candidemia
**Candidemia**	0.99 (0.86 - 1.34)	Bacteremia, Mixed, Negative

*I.Q.* interquartile range.

**Table 5:** Procalcitonin values according to the classification of PCR results

**Group**	**PCT (ng/ml) Median (I.Q.)**	**Different (p < 0.05) from**
**Bacteremia**	16.54 (7.83 - 47.8)	Mixed, Candidemia
**Mixed**	6.08 (4.29 - 12.65)	Bacteremia, Negative, Candidemia
**Negative**	13.4 (9.36 - 41.5)	Mixed, Candidemia
**Candidemia**	1.27 (0.92 - 2.09)	Bacteremia, Mixed, Negative

*I.Q*. interquartile range.

**Figure 4:**
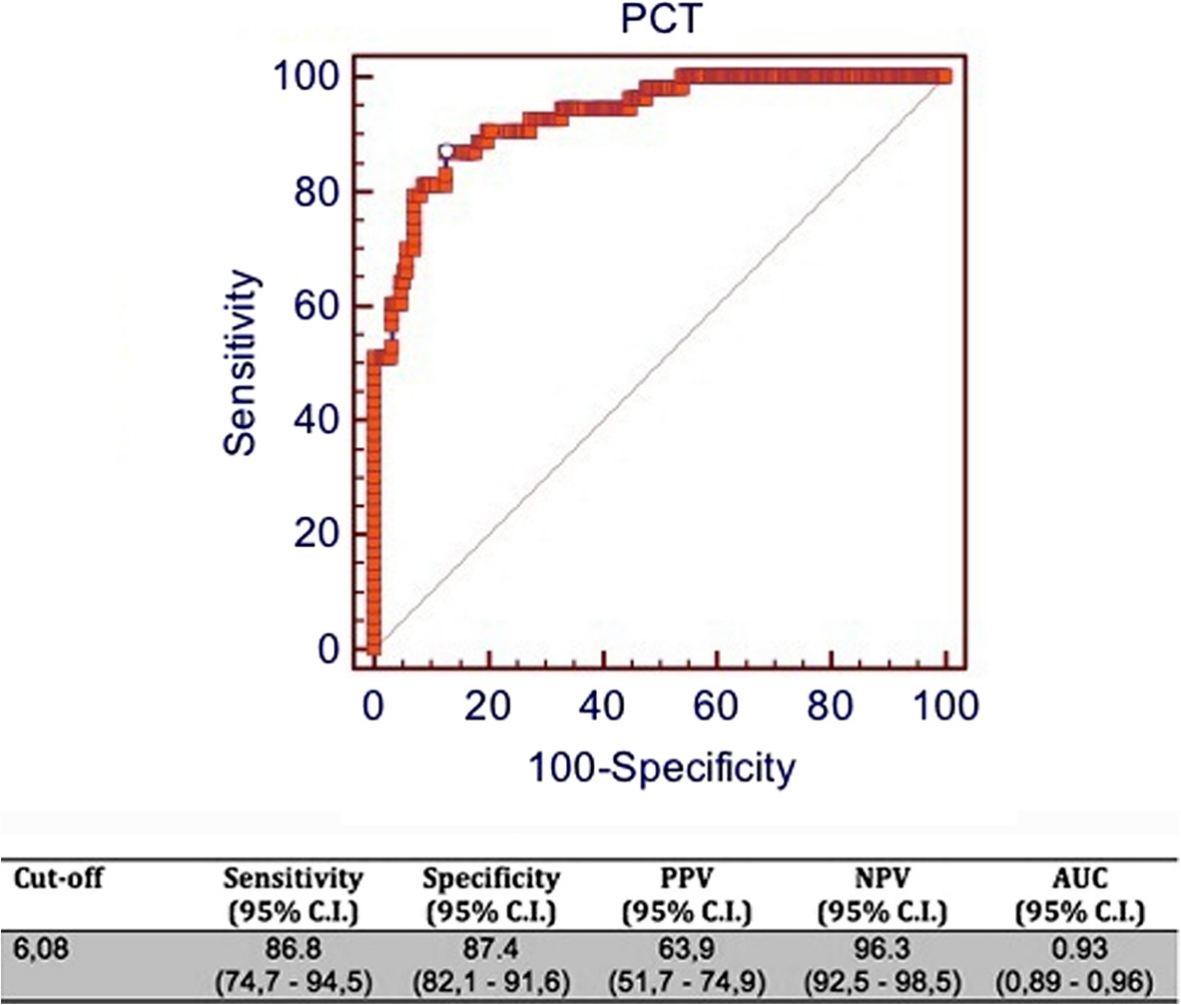
**ROC curve of PCT for prediction of identification of Candida spp. by BC.** C.I = confidence interval; PPV = positive predictive value; NPV = negative predictive value; AUC = area under the curve.

**Figure 5:**
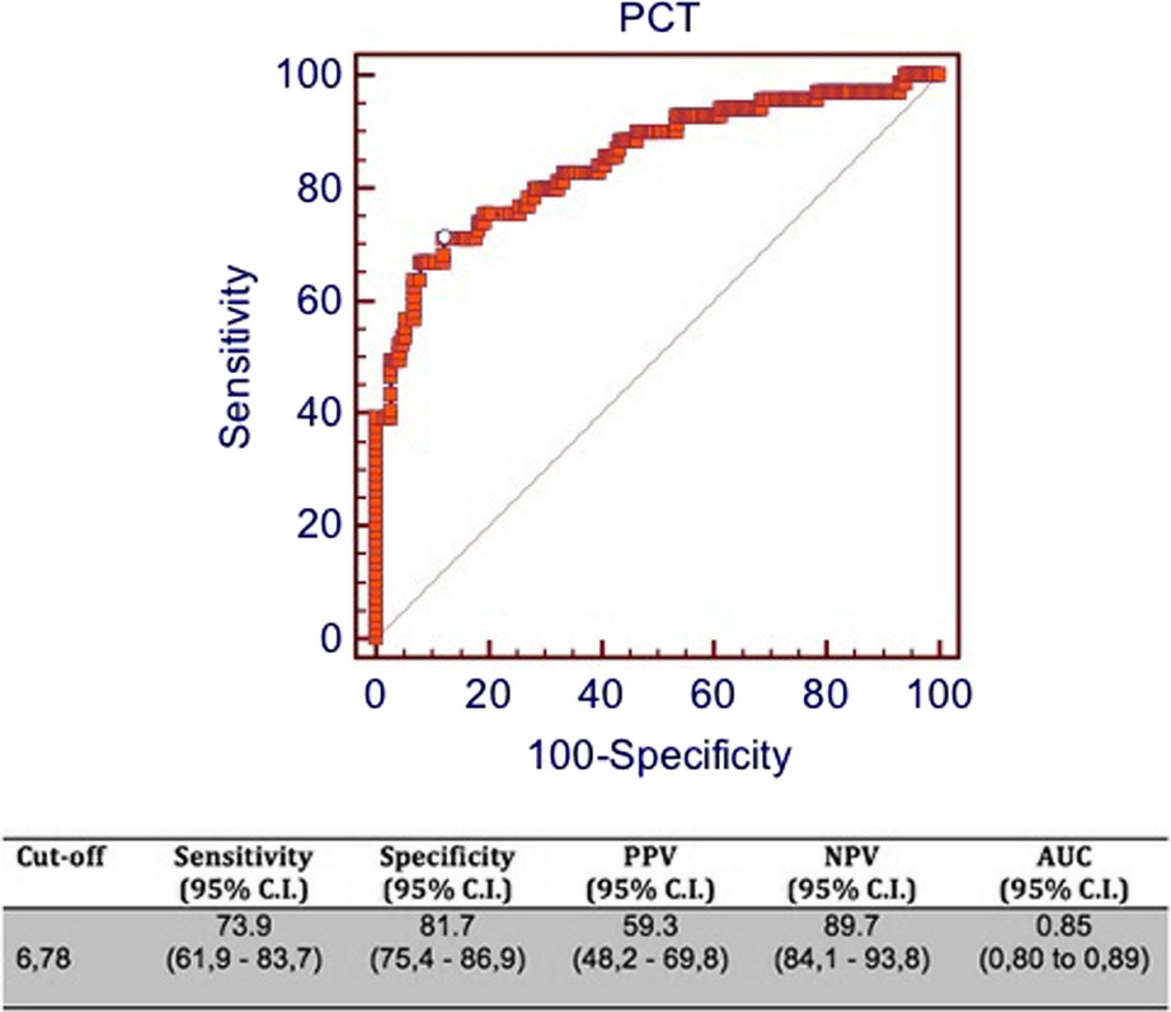
**ROC curve of PCT for prediction of identification of Candida spp. by PCR.** C.I = confidence interval; PPV = positive predictive value; NPV = negative predictive value; AUC = area under the curve.

A cut-off of 6.08 ng/ml for PCT showed a sensitivity of 86.8%, a specificity of 87.4%, a PPV of 63.9%, a NNP of 96.3% and an AUC of 0.93 for identification of Candida spp. (alone or in association with bacteria) by BC (Figure 4). Similarly, according to PCR results, a cut-off of 6.78 ng/ml yielded a sensitivity of 73.9%, a specificity of 81.7%, a PPV of 59.3%, a NPV of 89.7 and an AUC of 0.85 (Figure 5).

The variables included in the logistic regression model were the value of PCT and the subsequent diagnostic episodes, which both resulted independently associated with the presence of Candida spp. detected by either BC or PCR (Tables 6 and 7 respectively).

**Table 6:** Logistic regression Candidemia

**Variable**	**Variable type**	**β coefficient**	**Standard error**	***P value***
**PCT**	Continuos	-0.41	0.07	< 0.0001
**Subsequent Diagnostic episode**	Dichotomous	2.23	0.48	< 0.0001

Variables not included in the model: age, sex, SOFA score, Surgical, WBC, Septic Shock. (Hosmer & Lemeshow test: chi-square: 4.49, p = 0.81).

**Table 7:** Logistic regression PCR

**Variable**	**Variable type**	**β coefficient**	**Standard error**	***P value***
**PCT**	Continuos	-0.07	0.016	< 0.0001
**Subsequent Diagnostic episode**	Dichotomous	1.38	0.33	< 0.0001

Variables not included in the model: age, sex, SOFA score, Surgical, WBC, Septic Shock. (Hosmer & Lemeshow test: chi-square: 99.7, p < 0.0001).

The different Candida spp. isolated by BC and PCR are shown in Tables 8 and 9.

**Table 8:** Candida spp. detected (according to BC results) and their PCT values (median)

**Candida spp**	**Diagnostic episodes (%)**	**PCT value Median (I.Q.)**
**C. Albicans**	13 (59.0)	1.32 (0.92 - 2.0)
**C. non albicans**	9 (41.0)	0.92 (0.68 - 1.98)
**C. Parapsilosis**	6 (27.3)	0.90 (0.83 - 1.29)
**C. glabrata**	1 (4.5)	2.98
**C. krusei**	2 (9.1)	1.58 (0.12 - 3.05)
**C. tropicalis**	0	

*I.Q.* interquartile range.

Albicans vs non albicans p = 0.36.

**Table 9:** Candida spp. detected (according to PCR results) and their PCT values (median)

**Candida spp**	**Diagnostic episodes (%)**	**PCT value Median (I.Q.)**
**C. Albicans**	14 (46.7)	1.32 (0.97 - 2.29)
**C. non albicans**	16 (53.3)	1.08 (0.86 - 1.60)
**C. Parapsilosis**	10 (33.3)	0.92 (0.84 - 1.20)
**C. glabrata**	3 (10)	2.09 (1.21 - 2.98)
**C. krusei**	2 (6.7)	1.58 (0.12 – 3.05)
**C. tropicalis**	1 (3.3)	1.56

*I.Q.* interquartile range.

Albicans vs non albicans p = 0.25.

PCT values did not differ between Candida albicans and non-albicans species isolated by BC (p = 0.36) and PCR (p = 0.25).

## Discussion

A prompt start of effective antifungal therapy is essential for mortality reduction in Candida spp. BSI [4,5]. Even if BC is the cornerstone for diagnosis of Candida spp. BSI, concern has been raised about the long time required before microbiological response [28,29]. A pre-emptive approach aims to start treatment without delay in patients with clinical and microbiological evidence of candidemia without its definitive BC-based proof, while limiting the use of antifungal agents in low-risk patients [9,28,30,31]. Surrogate markers of candidemia were studied for this purpose. β-1,3–D – glucan is not specific for Candida spp. BSI as it is considered a panfungal diagnostic method [7,14]. Despite a high negative predictive value, different patient characteristics (i.e. hematological diseases) and interventions (i.e. albumin or immunoglobulin administration, hemodialysis) are responsible for its reported low specificity [7,11,13,14,32]. The use of surgical gauzes containing glucan may increase β-1,3–D – glucan level leading to false positive results and to its poor reliability in ICU with a high admission rate of surgical patients [12,33,34]. The combined determination of mannan and anti-mannan antibodies in serum is a specific diagnostic marker of Candida spp. detection with a high negative predictive value [10,15,35,36]. Serial determinations are needed to optimise the diagnostic performance of both β-1,3–D – glucan and mannan/anti-mannan antibodies leading to the high related costs. Concerning PCR techniques as diagnostic tool for Candida spp. BSI, one of the most important limits precluding the widespread adoption is the potential contamination with clinically not significant fungal DNA [16]. Moreover, PCR-based molecular techniques require technical skills and equipment. Further studies are needed to assess their diagnostic performance and harmonization of results according to the adopted PCR-technique [7,16]. Few data support the usefulness of PCT as surrogate marker of Candida spp. BSI. [22-24,37] and no studies have investigated PCT values in patients with BSI caused by both Candida spp. and bacteria. Moreover, the diagnostic performance of PCT has been investigated in light of the BC results and not considering the results of a real-time PCR technique. In this study, we tested PCT as surrogate marker of identification of Candida spp. by BC or PCR whether alone or in association with bacteria in septic patients. We identified a cut-off for PCT level useful to rule out the presence of Candida spp. with a high negative predictive value. The finding of a significantly lower PCT level in Candida spp. BSI than in bacterial BSI is consistent with that previously reported [22-24]. According to our data, in BSI caused by both Candida spp. and bacteria, the PCT showed a higher level than in BSI caused only by Candida spp. but a lower level than in bacterial and negative BSI. Of note, Candida spp. infection was diagnosed as a second BSI in a relevant number of cases, confirming previously published data [23]. This finding may be explained in light of the novel recognition of sepsis as a complex immune response to pathogens with a hyperinflammatory phase as a hallmark of the early period followed by a phase of impaired immunity and an anti - inflammatory phase predominance. Due to this susceptibility, a secondary nosocomial infection such as Candida spp. infection, typically occurs [38-41]. It may be hypothesized that the observed low PCT level in relation to Candida spp. infection may reflect this impaired immunity phase of sepsis or rather may result from a different inflammatory pattern stimulated by fungal infection [23,42]. Indeed, a lower level of PCT was observed in patients with a subsequent BSI than in those experiencing the first BSI, regardless of the identification of Candida spp. as the responsible organism for the subsequent BSI [42]. Thus, identification of PCT level in the case of a mixed infection is important since intensivists encounter a growing number of critically ill patients in immunoparalysis prone to Candida spp. infection and a promptly anti-fungal therapy institution is pivotal for mortality reduction [4,38]. Indeed, a high number of Candida spp. are detected in association with a bacterial infection, namely by gram negative species, in daily practice of our ICU with its high number of surgical patients. The high negative predictive value of PCT for detection of Candida spp. by either BC or PCR may represent a useful tool to exclude the presence of candidemia and guide the antifungal treatment regimens in critically ill patients, with the advantage, among others, of costs reduction. [43] In our opinion, the limitations of the study are its retrospective design and the low number of Candida spp. BSI analysed, which reflects the low, though increasing, prevalence of Candida spp. BSI [3,44,45]. Another limitation may originate from the characteristic epidemiology of pathogens causing BSI in our general ICU with a high admission rate of surgical patients. Among them we observe the predominance of the abdominal surgical patient who frequently underwent an emergency procedure. This setting leads to the high prevalence of Gram negative pathogens in our population as responsible for BSI, either alone or in association with Candida spp. [1,46,47]. However, patients with complicated abdominal surgery (i.e. secondary peritonitis) are at high risk of Candida spp. BSI and this population would benefit from a validated marker for this kind of infection [48]. Previous studies reported a higher level of PCT for Gram negative BSI than in patients with a BSI caused by a Gram positive infection, leading to the conclusion that PCT may represent a useful tool in differentiating Gram positive from Gram negative BSI. Nevertheless, this study was not designed to reach this goal. In our opinion, it is unlikely that Gram positive bacteria could have influenced PCT values both in bacterial and mixed BSI, given their low prevalence. Finally, different data show the higher sensitivity of home-made PCR compared to non home-made PCR techniques for fungal species detection [17]. The lower diagnostic performance of PCT - analysed for PCR results prediction (Figure 5) - may be explained by the lower specificity for living yeast since PCR technique aims at identifying solely pathogen DNA molecules. Nevertheless, BC is still considered the gold standard for comparison of alternative diagnostic methods [7] and our results may be interpreted in light of BC findings.

## Conclusions

According to our data, PCT determination represents a useful diagnostic tool to exclude the presence of Candida spp. in BSI of critically ill patients. It may be adopted as a component of a diagnostic strategy aiming to limit the unwarranted use of antifugal agents.

### Key messages

• Procalcitonin shows a high negative predictive value for detection of Candida spp. by blood culture or polymerase chain reaction either alone or in a mixed infection with bacteria.

• Procalcitonin may represent a useful tool for the diagnostic workup of critically ill patients with Candida bloodstream infections.

## Abbreviations

AUC: Area under the curve; BC: Blood culture; BSI: Blood stream infection; ICU: Intensive care unit; IR: Interquartile range; NPV: Negative predictive value; PCR: Polymerase chain reaction; PCT: Procalcitonin; PPV: Positive predictive value; ROC: Receiver operating characteristic; SD: Standard deviation; SOFA: Sequential organ failure assessment; SPP: Species; WBC: White blood count.

## Competing interests

The authors declare that they have no competing interests.

## Authors’ contributions

AC conceived the study, participated in data collection, performed the statistical analysis and draft the manuscript; VR participated in the design of the study, in data collection and draft the manuscript; FM participated in data collection and draft the manuscript; GF participated in data collection and draft the manuscript; GA participated in data collection and draft the manuscript; CP participated in data collection and revised the manuscript adding important intellectual content; SMR participated in the design of the study and in data collection; AG conceived the study and revised the manuscript adding important intellectual content. All authors read and approved the final manuscript.

## Pre-publication history

The pre-publication history for this paper can be accessed here:

http://www.biomedcentral.com/1471-2253/14/9/prepub
